# ACERT: Agent‐Based Model of Complex Life Cycle Evolution—R Tools

**DOI:** 10.1002/ece3.74240

**Published:** 2026-09-15

**Authors:** Jesus A. Lopez, Robert B. Page, Ashley I. Teufel

**Affiliations:** ^1^ Department of Natural Sciences Texas A&M University–San Antonio San Antonio Texas USA; ^2^ Department of Biology & Microbiology Northwestern State University Natchitoches Louisiana USA

**Keywords:** agent‐based modeling, complex life cycles, metamorphosis, population genetics, simulation analysis

## Abstract

Agent‐based models (ABMs) are increasingly used to study eco‐evolutionary dynamics in organisms with complex life cycles, but downstream analysis of model output remains a bottleneck. Simulations can generate millions of records across individuals, time points, and replicates, often spread across many files and requiring extensive custom preprocessing before biological interpretation is possible. We present ACERT (Agent‐based model of Complex life cycle Evolution—R Tools), an open‐source R package that provides an integrated workflow for importing, cleaning, analyzing, and visualizing ABM outputs tailored to systems with complex life cycles. ACERT reads individual‐level records for mobile agents, called “turtles” in NetLogo terminology, patch‐level environment data, and allelic mutation files; standardizes these streams into analysis‐ready structures; and links directly to established population‐genetic inference methods. The package supports phenotype and demographic summaries, spatial and landscape diagnostics, migration and replicate similarity assessment, population‐genetic diversity and differentiation statistics, and exploratory microevolutionary tools including major‐allele trajectory tracking and selection‐screening workflows. ACERT also includes functions for generating synthetic example datasets and building reproducible reports. By consolidating domain‐specific preprocessing and analysis steps into a single, coherent package, ACERT reduces the technical overhead associated with simulation‐based inference and improves reproducibility.

## Graphical abstract

ACERT is an open‐source R package that closes the gap between raw agent‐based model output and biological interpretation for systems with complex life cycles. It provides a single, coherent pipeline for data preprocessing, population‐genetic analysis, spatial diagnostics, and replicate quality control, eliminating the custom scripting bottleneck that limits reproducibility in simulation‐based eco‐evolutionary research.

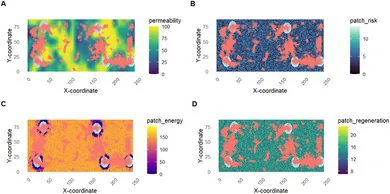

## 1 Introduction

Organisms with complex life cycles occupy a special and challenging position in evolutionary ecology. Species such as amphibians, many insects, and a range of marine invertebrates pass through discrete developmental stages that differ in morphology, physiology, resource use, habitat, and the selective pressures they experience (Wilbur 1980). In amphibians, for example, an aquatic larval phase and exposure to aquatic predators give way through metamorphosis to a terrestrial adult phase in which locomotion, foraging strategy, and the relevant abiotic stressors are entirely transformed. Selection can therefore act differently across stages of the same life cycle, leading to genetic architectures and eco‐evolutionary feedbacks that are difficult to disentangle with observational data alone (Moran 1994). Understanding how genotypes, phenotypes, and population structure evolve jointly under these conditions requires tools that can represent individual variation, spatial heterogeneity, and developmental stage transitions simultaneously.

Agent‐based models (ABM) provide a natural computational framework for studying complex life cycle evolution because they represent heterogeneous individuals as discrete agents whose state variables, behavioral rules, and fitness consequences can differ by developmental stage, location, and local environment (Grimm et al. 2005; DeAngelis and Mooij 2005). An ABM can track genotypes through successive breeding events, propagate allele frequencies across a spatially explicit landscape, couple life‐history outcomes at metamorphosis to both larval experience and adult environment, and replicate this entire process across hundreds of stochastic simulation runs. This capacity for mechanistic, individual‐level representation makes ABMs attractive for questions that sit at the intersection of landscape ecology, population genetics, and life‐history theory.

Realizing the full inferential potential of such models, however, requires bridging the gap between raw simulation output and biological interpretation. A typical ABM of complex life cycle evolution might produce hundreds of large files per simulation experiment, each containing individual‐level records for tens of thousands of agents across dozens of time steps, alongside patch‐level environmental conditions and a separate log of allelic mutations. Transforming these raw outputs into the data structures required by population‐genetic inference packages such as the genind objects expected by adegenet (Jombart 2008) or the allele frequency tables needed by hierfstat (Goudet 2005) demands substantial custom scripting. Beyond the population‐genetic layer, ecological summaries of life‐stage demography, spatial agent density, migration, and replicate‐level variation each require their own bespoke pipelines, compounding the burden.

Agent‐Based Model of Complex Life Cycle Evolution—R Tools (ACERT) was developed to close this gap. The package provides a coherent end‐to‐end workflow that begins with raw simulation output files and culminates in publication‐ready visualizations and population‐genetic summaries, without requiring users to write extensive custom parsing code for each analysis. Importantly, ACERT does not attempt to replace established statistical methods; instead, it standardizes and reorganizes simulation output so that existing, well‐validated R packages can be applied efficiently and reproducibly. This philosophy leverages the collective development effort invested in upstream population‐genetic and statistical packages and makes ACERT's outputs directly interpretable within the broader literature.

ACERT processes output from a spatially and genetically explicit ABM developed to study amphibian complex life cycle evolution. In this model, individual agents follow a biphasic life cycle modeled on species such as *Ambystoma maculatum* , progressing through egg, larval, juvenile, and adult stages on a landscape of discrete patches whose properties, including hydroperiod, resource availability, permeability, and predation risk, can be parameterized from user‐specified inputs or imported directly from GIS surfaces. Each agent carries a diploid genome of 10 loci that segregate in Mendelian fashion and contribute additively to metamorphic risk, linking individual genotype to the timing of the larval‐to‐juvenile transition. Key physiological state variables including stored energy reserves (t.energy), stress (t.stress), and energy consumed per time step (t.energy.consumption) are updated at each time step as functions of both intrinsic organismal state and local patch conditions, creating the individual‐by‐environment interactions that drive emergent population‐level dynamics. Simulation experiments are typically run across dozens of stochastic replicates and hundreds of time points, producing the high‐dimensional individual‐agent, environment, and mutation output files.

## 2 Software Scope and Design Philosophy

ACERT is organized around three primary data streams generated by the ABM framework. Turtle data, using NetLogo's (Wilensky 1999) term for individual mobile agents, capture the state of individuals—their spatial coordinates, life stage, sex, phenotypic measurements, developmental transitions such as age at metamorphosis, and multilocus genotypes encoded in a model‐specific chromosome format. Environment data describe patch‐level conditions at each time step, including resource availability and habitat quality. Mutation data provide a longitudinal record of allelic changes through simulation time, linking specific loci to the agents in which new mutations arose and enabling reconstruction of allele‐frequency trajectories across the experiment.

ACERT treats stochasticity as a property of the originating ABM rather than as a process imposed by the package. Depending on the model, variation among replicates may arise from demographic events such as survival, movement, and reproduction; environmental variation through time or space; and genetic processes such as segregation, mutation, and drift. ACERT preserves replicate identifiers across its data streams so that users can summarize and compare the resulting variation, but it does not generate, remove, or otherwise modify the stochastic processes represented by the originating simulation.

The design of the package reflects a set of priorities that emerged from repeated experience analyzing large, multi‐replicate simulation experiments. First, scalable data intake is essential; even a modestly sized experiment may involve hundreds of output files per data stream, and reading them serially with base R import functions is prohibitively slow. Second, the transformations required to move from raw simulation output to analysis‐ready data structures are complex enough that they should be handled by tested, documented functions rather than reproduced ad hoc in user scripts. Third, the package should be modular: a user interested only in spatial patch diagnostics should not need to run population‐genetic pipelines first, and a user focused on replicate‐level quality control should be able to do so without loading genotype data. Finally, the defaults and function signatures should remain accessible to researchers comfortable with base R and tidyverse‐style workflows, even if the underlying computations draw on more specialized packages.

## 3 Implementation and Architecture

**FIGURE 1:**
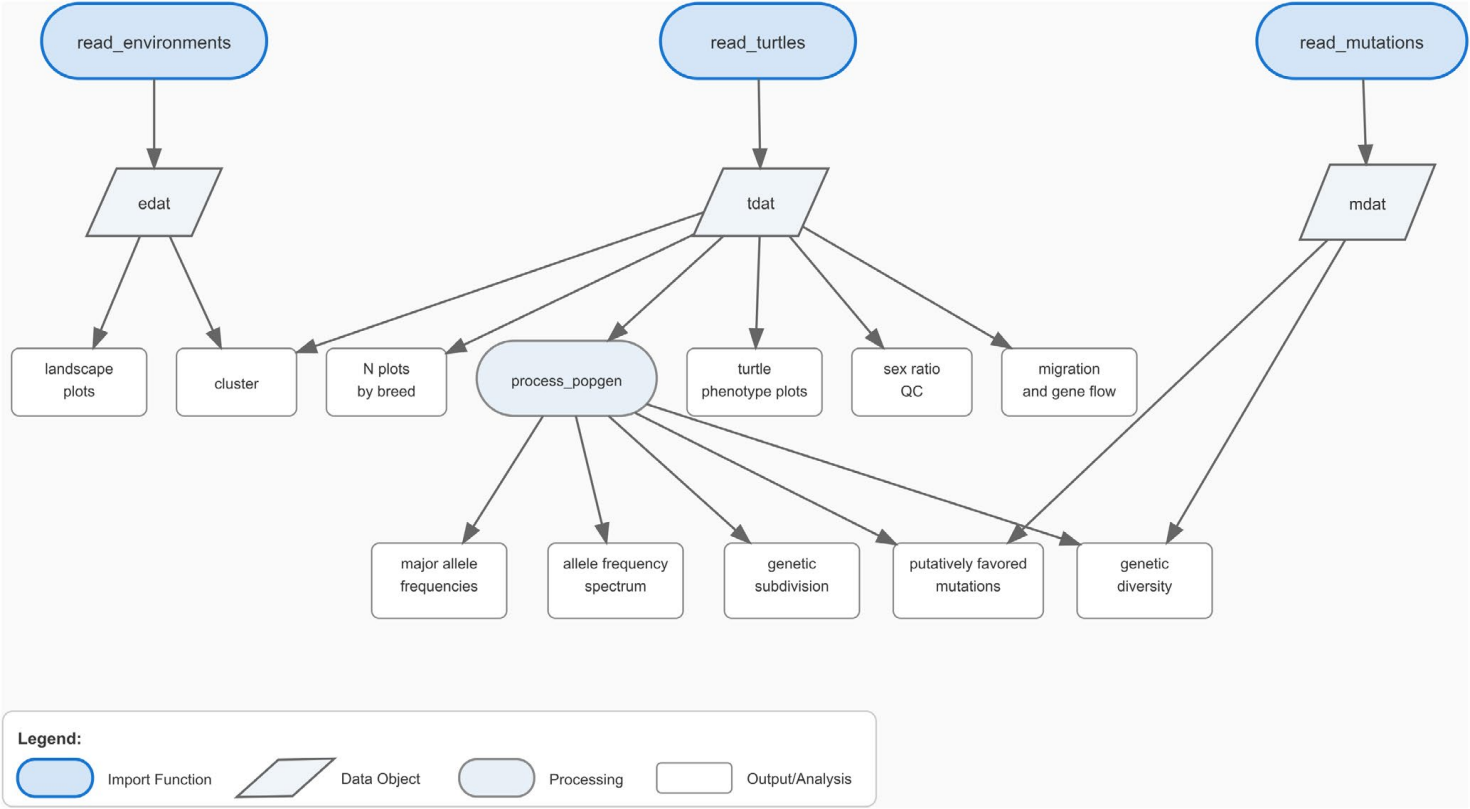
Data processing pipeline in ACERT. Turtle, environment, and mutation data are imported from raw simulation files and routed through preprocessing modules before branching into dedicated analysis pathways for population‐genetic summaries, spatial and landscape diagnostics, replicate quality control, and report generation. Arrows indicate the recommended flow, but most modules can be used independently once data are in the standardized internal format.

ACERT (version 0.1.0, GPL‐3) is implemented in R and organized into domain‐focused modules that correspond to the major analytical tasks encountered in complex life‐cycle simulation research (Figure 1). Core computational dependencies include dplyr and tidyr for tabular transformation, and ggplot2 (Wickham et al. 2019) for visualization. Population‐genetic analyses draw on adegenet (Jombart 2008), hierfstat (Goudet 2005), pegas (Paradis 2010), and poppr (Kamvar et al. 2014).

### 3.1 Data Input and Preprocessing

The import layer exposes three primary functions: read_turtles(), read_environments(), and read_mutations(), each of which reads all files matching a user‐specified pattern within a directory. This design means that adding a new simulation replicate to an analysis requires only placing the output file in the appropriate directory; no manual bookkeeping of run labels is needed. For turtle data specifically, the import function handles the model‐specific chromosome encoding scheme, in which multilocus genotypes are stored as delimited strings within a single column, and standardizes column types so that downstream functions receive consistently typed inputs regardless of minor variation in model output formatting. ACERT is designed for NetLogo output, and specifically for the schema exported by the focal model. It is not a multi‐platform adapter, and the readers do not auto‐detect arbitrary simulation output. In this respect ACERT occupies a position analogous to platform‐specific post‐processing packages such as HexSimR (Pacioni et al. 2018), for HexSim (Schumaker and Brookes 2018) output. Output from NetLogo models using different variable names, or from other simulation platforms, must be mapped onto the expected column names and chromosome encoding before import; for NetLogo models this is generally limited to renaming columns and, where genotypes are tracked, re‐encoding them into the delimited chromosome‐string format expected by read_turtles(). Within this schema, turtle, environment, and mutation files from each replicate can be stored in a common directory and distinguished using the file‐pattern arguments. Table 1 summarizes this directory structure and lists the expected headers for each data stream.

**TABLE 1:** Directory organization and expected column headers for ACERT input files from the focal model. Files from each replicate may be stored in a common directory and distinguished using the file‐pattern arguments.

Component	Required organization or fields
Directory layout	
Folder	sim_output/
Replicate 1	1_turtles.txt 1_environment.txt 1_mutations.txt
Replicate 2	2_turtles.txt 2_environment.txt 2_mutations.txt
Expected column headers for the focal model	
Turtle	ticks who breed t‐age t‐age‐metamorphosis t‐birth‐pond‐index t‐current‐pond‐index t‐dad‐chromosome‐1 t‐dad‐chromosome‐2 t‐dad‐id t‐energy t‐energy‐consumption t‐hatch‐countdown t‐max‐size t‐metamorphic‐risk t‐mom‐chromosome‐1 t‐mom‐chromosome‐2 t‐mom‐id t‐sex t‐size‐cm t‐size‐cm‐metamorphosis t‐stress ycor xcor
Environment	ticks pxcor pycor patch_energy patch_risk permeability patch_regeneration new index form
Mutation	ticks turtle old_allele old_additivity_value new_allele new_additivity_value

The package repository includes example input files for all three data streams that users can compare against their own exported files before running a full analysis. Notably, the package operates downstream of model execution and does not communicate directly with NetLogo (Wilensky 1999) or RNetLogo (Thiele et al. 2012). Compatibility therefore depends on the structure of the exported files rather than on a particular version of either program. Following changes to NetLogo, RNetLogo, or a model's export procedures, users should confirm that turtle, environment, and mutation files retain the documented field names, delimiters, and chromosome encoding. For space‐formatted turtle files, the documented field order must also be retained. We recommend importing a small test output and inspecting the resulting column names and values before processing a complete simulation experiment. Files that do not conform to the documented input schemas should be reformatted before import.

Because some turtle‐level variables are biologically meaningful only at specific life stages (e.g., metamorphic measurements) and are undefined for individuals that have not yet completed metamorphosis, preprocessing in ACERT applies explicit stage‐aware missing‐value rules through apply_breed_na_rules(). This function encodes the biologically appropriate treatment of absent values for each variable, preventing analytical errors such as inadvertently carrying forward larval measurements into adult‐stage summaries or averaging across life stages where pooling is not meaningful. Additional preprocessing utilities include functions for chromosome structure validation, allele extraction from encoded strings, and format conversion to the data structures expected by downstream population‐genetic packages. For large datasets, all three import functions support row sampling and time‐point subsetting, and the turtle and environment readers also support optional parallelization across files. A practical exploratory workflow is to inspect a small sample, restrict the import to biologically relevant time points, and then repeat final analyses on the complete data or on multiple sampling intensities to confirm that conclusions are not sensitive to data reduction.

### 3.2 Population‐Genetic Workflows

A central use case for ACERT is the extraction and analysis of population‐genetic signals from simulation output. The function process_popgen() serves as the primary interface for this workflow. It takes a cleaned turtle data frame, a vector of locus names, and an optional max_individuals limit for subsampling large replicates and returns genind objects. The genind format is the common currency of several major population‐genetics packages in R, so producing it directly from simulation output allows users to apply any compatible method without further data transformation.

Building on this foundation, ACERT provides wrappers for the most common inferential tasks encountered in simulation‐based population‐genetic research. The calculate_genetic_diversity() function computes per‐locus and per‐population observed heterozygosity, expected heterozygosity under Hardy–Weinberg equilibrium, and effective allele counts, offering a compact summary of within‐population variation that can be tracked across simulation time or compared across experimental treatments. Differentiation between populations is quantified through $F_{\mathrm{ST}}$ (Paradis 2010), Nei's $G_{\mathrm{ST}}$, Hedrick's standardized $G_{\mathrm{ST}}^{\prime }$, and Jost's D (Jost 2008), all of which can be computed pairwise to produce distance matrices suitable for visualization or further multivariate analysis. ACERT wraps the analysis of molecular variance (AMOVA) implementation in poppr to partition genetic variance (Excoffier et al. 1992). A discriminant analysis of principal components (DAPC) wrapper facilitates exploratory clustering of genotypes, which is useful for identifying whether simulated populations have diverged in multivariate genotype space across landscape configurations or experimental treatments (Jombart 2008).

Complementing these summary statistics, ACERT includes tools for computing and plotting allele frequency spectra at user‐selected loci and time points. Because mutations in the ABM arise at specific loci and spread stochastically through the population, tracking the frequency trajectory of derived alleles across simulation time is an informative way to characterize the action of selection, drift, and migration. Functions for major‐allele trajectory visualization and selection‐screening workflows are included as exploratory tools. The selection‐screening function uses linear regression to identify alleles with significant positive frequency trends and cross‐references those alleles with the mutation log.

### 3.3 Spatial, Landscape, and Demographic Diagnostics

**FIGURE 2:**
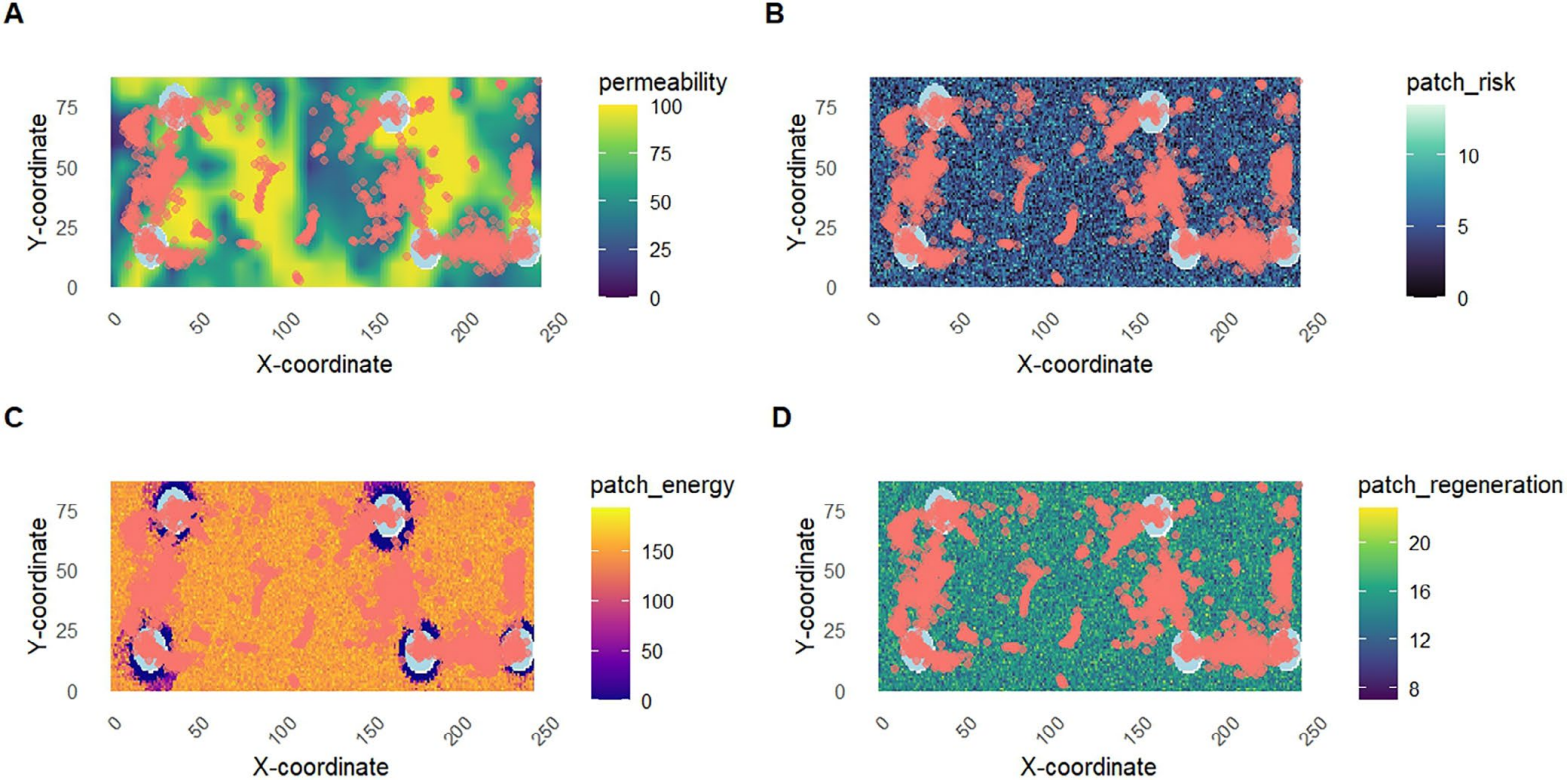
Example output of plot_netlogo_landscape() showing spatial variation in patch‐level environmental properties across the simulated landscape. Each panel displays a single patch property mapped across X‐ and Y‐coordinates, with salmon‐colored regions indicating individual agents. (A) Landscape permeability, reflecting the ease of movement through terrestrial habitat. (B) Patch risk, representing the local predation or hazard level experienced by agents. (C) Patch energy, indicating resource availability. (D) Patch regeneration rate, describing the capacity of patches to recover resources over time.

The environment data stream supports a complementary set of spatial analyses. plot_netlogo_landscape() produces maps of user‐selected patch properties at specified time steps, enabling rapid visual assessment of whether simulated landscapes vary as intended across treatment conditions and whether environmental gradients are maintained through simulation time (Figure 2). Turtle density across patches is computed by calculate_turtle_density(), which can be applied after subsetting the turtle data by life stage, sex, or genotype class, supporting questions about habitat preference, stage‐specific space use, and density‐dependent regulation. Because ponds are the focal breeding and larval‐development units in complex‐amphibian life‐cycle models, ACERT includes coordinate‐based nearest‐neighbor matching to assign individual turtles to their closest pond, enabling pond‐level summaries of demographic and genetic outcomes that map directly onto the spatial structure of the simulated landscape.

Demographic and phenotypic summaries are handled by analyze_turtle_property(), a general‐purpose function that computes group‐level statistics for any turtle‐level variable across user‐specified groupings. Common applications include comparing size at metamorphosis across birth ponds, tracking cohort‐level survival from hatching through metamorphosis, and monitoring the temporal trajectory of life‐history traits. Plotting wrappers generate figures for these summaries with minimal additional code, which is particularly useful when iterating across many parameter combinations. Sex ratio dynamics, which are ecologically informative in systems where sex determination or sex‐biased mortality interact with environmental conditions, are handled by a dedicated calculate_sex_ratio() function that can be applied across ponds, time steps, or experimental treatments.

### 3.4 Replicate Similarity and Quality Control

Stochastic ABMs are commonly run with multiple replicates to characterize variation among outcomes obtained under the same parameter set. ACERT provides an exploratory summary of this variation through assess_replicate_similarity(). For each user‐selected turtle property, the function calculates the mean and variance within each replicate. When environment data are supplied, it also calculates replicate‐level means and variances for patch energy, patch risk, permeability, and patch regeneration. The resulting turtle and environmental summaries remain available as separate columns in the returned replicate‐summary table.

For the clustering analysis, summary columns that are entirely missing or have no variance are removed, and the remaining columns are standardized and combined into a single feature matrix. ACERT calculates Euclidean distances among replicates, performs Ward hierarchical clustering, and evaluates k‐means partitions for values of k from 2 through the smaller of 10 or one fewer than the number of replicates. Mean silhouette scores are used to compare the tested partitions. The function returns the replicate summaries, distance matrix, hierarchical clustering, k‐means results, silhouette scores, reported optimal value of k, and corresponding cluster assignments. A companion plotting function can display the results as a dendrogram, principal‐component plot, or silhouette‐score plot. Genotype and mutation summaries are not included in this analysis.

These results are descriptive and do not constitute a formal test for anomalous replicates. The function does not automatically flag replicates, determine that a cluster represents invalid simulations, or recommend excluding any run. Because the tested k‐means solutions begin at k=2, the presence of multiple returned clusters should not by itself be interpreted as evidence of meaningful differences among replicates. Cluster assignments should instead be considered alongside the replicate summaries, distances, visualizations, and expected behavior of the originating model. Table 2 summarizes the objects returned by the function.

**TABLE 2:** Primary objects returned by assess_replicate_similarity(). These outputs support exploratory comparison of simulation replicates but do not identify invalid runs automatically.

Returned object	Contents
replicate_summaries	Replicate‐level means and variances for the turtle and environmental properties included in the analysis.
distance_matrix	Euclidean distances among replicates calculated from the standardized summary variables.
hclust	Ward hierarchical clustering of the replicate distance matrix.
kmeans_results	k‐means partitions for each tested value of k.
silhouette_scores	Mean silhouette score for each tested k‐means partition.
optimal_k and optimal_clusters	Reported highest‐scoring tested value of k and its corresponding replicate assignments.

### 3.5 Reporting and Data Export

create_abm_report() generates a structured output directory containing a standardized set of summary figures and a text summary derived from turtle, environment, and mutation data. The report is intended as a first‐pass diagnostic: a user who has just finished a batch of simulation runs can call this function to produce an overview of demographic outcomes, spatial patterns, and genetic summary statistics without writing any additional analysis code. Within create_abm_report(), the sample_for_plots argument defaults to TRUE. When the turtle dataset exceeds max_plot_points, which defaults to 50,000 records, the function randomly samples that number of records for the turtle‐property visualizations while leaving the supplied data object unchanged. The report also applies fixed internal limits to selected computationally intensive components: population‐genetic analyses use at most 100,000 turtle records and up to 1000 sampled records per replicate, mutation plots use at most 100,000 mutation records, and replicate‐similarity assessment uses at most 50,000 turtle records. Because these components use random sampling, users should call set.seed() before report generation when exact reproducibility is required. save_abm_data() supports export of processed data objects in CSV, RDS, tab‐delimited text, and other common formats, facilitating interoperability with other workflows.

Reproducibility is further supported by a testthat test suite that covers data import, preprocessing, spatial analysis, plotting, utilities, and population‐genetic helper functions, using fixture‐based test data that can be regenerated with the package's synthetic dataset functions. An executable vignette demonstrates an analysis workflow from synthetic replicate generation through population‐genetic and visualization outputs, providing a template that users can adapt to their own simulation experiments.

## 4 Minimal Workflow Example

A typical ACERT analysis begins with reading and combining all output files for a simulation experiment, followed by stage‐aware preprocessing, exploratory quality control, and then substantive phenotypic and genetic analyses. The following example illustrates this sequence; a more detailed walkthrough with annotated outputs is available in the package vignette.library(ACERT)# Import all output files from a simulation directorytdat <- read_turtles(path = "sim_output", pattern = "turtles.txt")edat <- read_environments(path = "sim_output", pattern = "environment.txt")mdat <- read_mutations(path = "sim_output", pattern = "mutations.txt")# Apply stage-aware missing-value rules to turtle datatdat_clean <- apply_breed_na_rules(tdat)# Assess replicate similarity across key life-history traitssim_diag <- assess_replicate_similarity(    turtle_data = tdat_clean,    env_data    = edat,    properties  = c("t.age.metamorphosis",                   "t.size.cm.metamorphosis",                   "t.stress"))# Subset to adult turtles at the final time step for genetic analysisfinal_adults <- subset(tdat_clean,                       breed == "adults" & ticks == max(ticks)) # Build genind objects and per-locus FST summariespg <- process_popgen(final_adults,                     loci_names = letters[1:10],                     progress   = FALSE)# Compute per-population genetic diversity statisticsdiv <- lapply(pg$genind_data, calculate_genetic_diversity)# Generate a structured report directoryreport_dir <- create_abm_report(tdat_clean, edat, mdat,                               output_dir = "results") 


In this workflow, read_turtles() reads and combines all files in the sim_output directory whose names match the specified pattern, appending a run identifier to each row. apply_breed_na_rules() enforces life‐stage‐appropriate missing‐value conventions before any summaries are computed. The subsequent subsetting to final‐tick adults isolates the time point and life stage most relevant to reproductive fitness in the model, and process_popgen() transforms the genotype columns of that subset into genind objects that are compatible with the full range of adegenet and hierfstat methods. create_abm_report() then writes a self‐contained output folder with standard diagnostic figures derived from all three data streams, providing a record of the analysis alongside the outputs themselves.

## 5 Use Cases

ACERT is intended for simulation studies in which life‐history traits, spatial environment, and genotypic composition evolve jointly under ecologically realistic conditions. One representative application is the comparative analysis of evolutionary outcomes across landscape configurations, for example, asking how the connectivity and spatial arrangement of breeding ponds influence the partitioning of genetic diversity within versus among ponds, or how variation in aquatic cycling affects the co‐evolution of larval growth rates and stress tolerance across the metamorphic boundary. ACERT's integrated pipeline from environmental patch summaries through population‐genetic differentiation statistics makes this kind of multilevel comparison straightforward.

A related application is the use of replicate distributions to characterize evolutionary stochasticity. When an ABM includes demographic, environmental, or genetic stochasticity, a key inferential question is often whether observed differences between parameter sets represent genuine treatment effects or fall within the range of between‐replicate variation expected under either condition. ACERT's replicate‐similarity and replicate‐level genetic summary tools allow users to build these distributions and to visualize them alongside point estimates, supporting more principled inference than reliance on single representative replicates.

The mutation‐tracking and allele‐trajectory components of ACERT support studies of microevolutionary dynamics. Tracking the frequency trajectory of specific alleles from introduction through fixation or loss allows users to identify alleles with sustained positive frequency trends and relate those patterns to mutation‐effect information recorded by the model. These tools are exploratory by design, providing a way to identify candidate loci for further formal analysis rather than serving as a substitute for model‐based inference.

## 6 Comparison to Existing Tools

Several existing R packages address adjacent parts of the simulation analysis pipeline but do not cover the full workflow that ACERT targets. Packages designed for interfacing with NetLogo, such as nlrx (Salecker et al. 2019) and RNetLogo (Thiele et al. 2012), provide tools for configuring and launching simulation experiments, managing parameter sweeps, and retrieving model output through a programmatic interface. Their focus is on experiment management and model execution rather than on the biological analysis of output, and they do not provide domain‐specific preprocessing for life‐stage structure, multilocus genotype extraction, or population‐genetic inference. A user working with a NetLogo‐based ABM of complex life‐cycle evolution would typically use nlrx for experiment management and then require a separate, custom analysis layer to connect raw output to population‐genetic packages, precisely the gap that ACERT addresses.

Within the population‐genetic analysis space, packages such as adegenet (Jombart 2008), pegas (Paradis 2010), hierfstat (Goudet 2005), and poppr (Kamvar et al. 2014) are mature and well‐documented, but they expect users to provide data already in specific formats such as genind, loci, or labeled allele‐frequency matrices. Constructing these objects from simulation output that encodes genotypes as model‐specific chromosome strings, and doing so consistently across hundreds of replicate files while preserving the run identifiers needed for replicate‐level analysis, is a nontrivial data engineering task. ACERT automates this transformation, acting as a specialized connector between ABM output and the existing population‐genetic toolchain rather than duplicating any of the inferential methods those packages provide.

The strataG package (Archer et al. 2017) offers a different approach to bridging simulation and inference, with tools for computing population‐genetic statistics, conducting coalescent simulations, and interfacing with external programs. It is focused primarily on empirical population‐genetic workflows; however, it does not address the import, preprocessing, or life‐stage‐aware cleaning requirements specific to individual‐based forward‐time simulation output. More broadly, no existing package in the R ecosystem targets the combined data management, spatial diagnostic, replicate quality‐control, and population‐genetic analysis needs that arise specifically from large‐scale ABMs of complex life‐cycle evolution.

## 7 Current Limitations and Future Development

Some computationally intensive analyses, particularly dense time‐series genetic tracking across all loci and replicates, can place substantial demands on memory and processing time. ACERT therefore supports sampling during import, restriction to selected time points, parallel turtle and environment import across files, and automatic sampling limits for selected report components. For analyses that still exceed available memory, users can process replicates sequentially and retain only the resulting summaries. Because reducing temporal resolution, loci, or individuals can obscure rare events or distort frequency estimates, results based on subsampling should be compared across sampling intensities and, when feasible, confirmed using the complete dataset.

Planned future directions include the development of animation tools for visualizing evolutionary and demographic dynamics through simulation time, tighter integration with the report generation system so that temporal animations can be included in automated output directories, and additional utilities for formal sensitivity analysis and parameter‐space exploration. Expanding the vignette library to cover a broader range of biological questions and model configurations will also improve the accessibility of the package to researchers new to simulation‐based inference in ecology and evolution.

## 8 Availability

ACERT is implemented in R and distributed under the GPL‐3 license. The source code, documentation, and vignette are maintained in the public repository at https://github.com/a‐teufel/ACERT, which is used as the primary release and distribution archive. Bug reports and feature requests can be submitted through the repository issue tracker at https://github.com/a‐teufel/ACERT/issues. The package can be installed directly from GitHub using devtools::install_github(“a‐teufel/ACERT”). The analyses described in this manuscript correspond to package version 0.1.0. The source code for the underlying amphibian complex life‐cycle agent‐based model is available separately at https://github.com/a‐teufel/Amphibian‐CLC‐Model.

## 9 Conclusion

Simulation‐based research on complex life‐cycle evolution has matured to the point where mechanistically realistic ABMs can track the joint evolution of life‐history traits, spatial population structure, and multilocus genotypes across biologically relevant timescales. Beyond the computational demands associated with large simulations, a major obstacle to realizing the full inferential potential of these models is the analytical gap between raw simulation output and the data structures and summary statistics needed to interpret results in a biological context. ACERT addresses this gap directly, providing a coherent, tested, and documented workflow that takes ABM output files as input and delivers population‐genetic summaries, spatial diagnostics, and replicate quality‐control assessments as output, with the flexibility to accommodate the full range of questions that arise in complex life‐cycle simulation research.

## Author Contributions

**Jesus A. Lopez:** conceptualization (equal), data curation (equal), formal analysis (equal), investigation (equal), methodology (equal), software (equal), validation (equal), visualization (equal), writing – original draft (equal), writing – review and editing (equal). **Robert B. Page:** conceptualization (equal), data curation (equal), formal analysis (equal), investigation (equal), methodology (equal), software (equal), supervision (equal), validation (equal), visualization (equal), writing – original draft (equal), writing – review and editing (equal). **Ashley I. Teufel:** conceptualization (equal), data curation (equal), formal analysis (equal), investigation (equal), methodology (equal), software (equal), supervision (equal), validation (equal), visualization (equal), writing – original draft (equal), writing – review and editing (equal).

## Conflicts of Interest

The authors declare no conflicts of interest.

## Data Availability

ACERT source code, documentation, and example workflows are available through the project repository at https://github.com/a‐teufel/ACERT.
